# Non-Vitamin K Antagonist Oral Anticoagulants and the Gastrointestinal Bleeding Risk in Real-World Studies

**DOI:** 10.3390/jcm9051398

**Published:** 2020-05-09

**Authors:** Larisa Anghel, Radu Sascău, Anca Trifan, Ioana Mădălina Zota, Cristian Stătescu

**Affiliations:** 1 Internal Medicine Department, “Grigore T. Popa” University of Medicine and Pharmacy, 700503 Iași, Romania; larisa.anghel@umfiasi.ro (L.A.); ancatrifan@yahoo.com (A.T.); madalina.chiorescu@gmail.com (I.M.Z.); cstatescu@gmail.com (C.S.); 2 Cardiology Department, Cardiovascular Diseases Institute, “Prof. Dr. George I.M. Georgescu”, 700503 Iași, Romania; 3 Gastroenterology and Hepatology Department, Gastroenterology and Hepatology Institute, 700019 Iași, Romania

**Keywords:** non-vitamin K antagonist oral anticoagulants, gastrointestinal bleeding, safety profile, real-world studies

## Abstract

In the present study, we aimed to provide evidence from high-quality real world studies for a comprehensive and rigorous analysis on the gastrointestinal bleeding (GIB) risk for non-vitamin K antagonist oral anticoagulants (NOACs). We performed a systematic search of MEDLINE, EMBASE and PUBMED, and of 286 records screened, we included data from 11 high-quality real-world studies, coordinated by independent research groups over the last 3 years, that reported major GIB events in patients given NOACs or vitamin K antagonists for patients with nonvalvular atrial fibrillation. The lowest risk of gastrointestinal bleeding was with apixaban compared with warfarin (hazard ratio (HR) for GIB for apixaban ranging between 0.45 (95% confidence interval (CI) 0.34 to 0.59) and 1.13 (95% CI 0.79 to 1.63)). Apixaban was associated with a lower risk of GI bleeding than dabigatran ((HR ranging between 0.39 (95% CI 0.27 to 0.58) and 0.95 (95% CI 0.65 to 1.18)) or rivaroxaban ((HR ranging between 0.33 (95% CI 0.22 to 0.49) and 0.82 (95% CI 0.62 to 1.08)). The results of our study confirm a low or a similar risk for major GIB between patients receiving apixaban or dabigatran compared with warfarin, and apixaban appears to be associated with the lowest risk of GIB.

## 1. Introduction

Since warfarin was approved for use in 1954, it has been the mainstay of anticoagulant treatment for patients with atrial fibrillation, deep vein thrombosis and pulmonary embolism. However, the use of vitamin K antagonists (VKAs) has many limitations, such as the need to ensure proper anticoagulation control by regular monitoring and also many interactions with drugs and diet. As a result, 30–50% of patients are undertreated [1,2,3,4]. In the last 10 years, the US Food and Drug Administration (FDA) has approved non-vitamin K antagonist oral anticoagulants (NOACs), long-awaited alternatives to the vitamin K antagonists that offer relative efficacy, safety and convenience. This new class of drugs includes dabigatran, rivaroxaban, apixaban and edoxaban. They have the convenience of fixed dosing with no need for laboratory monitoring or dietary discretion [5,6,7,8,9,10,11]. But despite these apparent advantages, debate still remains regarding the gastrointestinal bleeding (GIB) risk [12,13,14,15,16,17,18].

Compared with vitamin K antagonists or heparins, NOACs have rapid onset and offset of action, predictable pharmacodynamics, fewer food–drug and drug–drug interactions and can reversibly and directly inhibit a specific factor of the coagulation cascade [19,20,21,22,23] (Table 1).

Dabigatran targets thrombin and all xabans (apixaban, rivaroxaban, edoxaban) target factor Xa. The beginning of the anticoagulation effect is rapid and begins two hours following the first dose and is lost within 24 h after discontinuation of the drugs. Direct anti-Xa inhibitors are able to inhibit both free and prothrombinase-bound factor Xa, and may also be able to inhibit clot-associated factor Xa. It is important to mention that rivaroxaban does not react with antibodies implicated in heparin-induced thrombocytopenia [21,24,25].

Dabigatran is a direct thrombin inhibitor, administered as a prodrug (dabigatran etexilate) and absorbed principally in the stomach and proximal small bowel as an inactive prodrug. Serum and hepatic esterases metabolize this inactive prodrug to the active drug. The bioavailability of the drug is low, approximately 3–7%, with the unabsorbed dabigatran being converted to active dabigatran in the distal bowel and then excreted in the feces [25,26,27,28]. The majority of non-absorbed drug is excreted in the stool and the absorbed drug is mainly excreted unchanged by the kidneys. Compared with warfarin, which is not activated in the bowel, the active dabigatran in the distal bowel may promote gastrointestinal bleeding [25,26]. There are also studies reporting that dabigatran is associated with esophagitis and gastric ulceration, suggesting that the drug may directly injure the gastrointestinal mucosa [29]. Dabigatran may be given at a dose of 150 mg b.i.d or 110 mg b.i.d. The dose of 75 mg b.i.d can be administered in the presence of renal insufficiency (creatinine clearance (CrCl) < 50 mL/min). The drug is contraindicated in patients with severe renal impairment (CrCl < 30 mL/min) or advanced liver disease [30,31,32,33,34].

Apixaban is a direct inhibitor of factor Xa and has a bioavailability of 50%. Almost 25% of the absorbed drug is excreted by the kidney with a half-life of around 12 h. Apixaban is administered at a dose of 5 mg b.i.d, and 2.5 mg b.i.d if patients have at least 2 of the following features: age 80 years or older, body weight 60 kg or less, or serum creatinine 1.5 mg/dL or more [35,36,37,38,39,40].

Rivaroxaban is also a direct factor Xa inhbitior, with a bioavailability of 66% and a half-life that ranges from 6 to 13 h [35,41]. One-third of the absorbed drug is excreted by the kidney and two-thirds is metabolized by the liver into inactive forms. Rivaroxaban is administered at a dose of 20 mg daily, and 15 mg daily if the CrCl is <50 mL/min. The drug is also contraindicated in severe renal impairment (CrCl < 15 mL/min) and advanced liver disease [41,42,43,44,45].

The anticoagulant effect can be local and/or systematic and the sites of gastrointestinal bleeding differ for individual NOACs. They have a systemic anticoagulant effect and also a local effect such as: incomplete absorption (topical anticoagulant effect), direct caustic effect (tartaric acid in dabigatran) or inhibition of mucosal healing [24]. When compared with warfarin, aspirin or non-steroidal anti-inflammatory drugs, where upper gastrointestinal bleeding predominates [46], in the RE-LY trial, lower GIB was found in 53% of dabigatran users. In this post hoc analysis of the Randomized Evaluation of Long-Term Anticoagulation Therapy (RE-LY) trial, comparing dabigatran to warfarin, Kolb et al. [24] reviewed the cases of suspected GI bleeding. They collected data on the causative lesions and the site and acuity of bleeding within the bowel. They localized the bleeding in approximately two-thirds of the cases, and 47% of them were detected in the upper GI tract and 39% in the colon. It was interesting that the rate of bleeding in the upper GI tract was similar between the two doses of dabigatran studied (110 and 150 mg) as compared with warfarin, whereas lower GI bleeding (which included colonic, jejunal and ileal sources) was more frequent in patients treated with dabigatran as compared with warfarin (relative risk was 1.78 for dabigatran 110 mg and 2.23 for dabigatran 150 mg). This probably is related to the incomplete absorption of the active NOACs in the upper GI tract, which leads to an increased availability of dabigatran in the lower GI tract and also a topical effect on the mucosa leading to bleeding. This gastrointestinal bleeding risk is higher especially in the presence of preexisting lesions like angiodysplasias and erosions [47,48,49,50]. A recent study published by Contaldo et al. evaluated the gastrointestinal bleeding caused by NOACs with videocapsule endoscopy. They enrolled 109 patients with iron deficiency anaemia, 18 of them taking oral anticoagulants, and demonstrated a trend in the association of oral anticoagulant use with small intestinal lesions at videocapsule endoscopy, despite a non-statistical significance (odds ratio (OR) = 3.38; 95% confidence interval (CI) 0.73–15.70; *P* = 0.10). This study highlights the usefulness of videocapsule endoscopy in providing clear information in patients with unexplained iron deficiency anaemia [51].

Even if apixaban and rivaroxaban are both factor Xa inhibitors, with similar bioavailability, and are administered in active form, the risk of GIB differs in these two agents, and this may be related to the higher peak level of once-daily dosing of rivaroxaban than the twice-daily dosing of apixaban [52,53]. The risk factors for NOACs-related gastrointestinal bleeding are summarized in Table 2.

Considering the fact that debate still remains regarding the gastrointestinal bleeding risk for patients on anticoagulant therapy, either warfarin or NOACs, we aimed to highlight evidence from high-quality real world studies regarding the GIB risk for oral anticoagulants.

## 2. Experimental Section

The availability of warfarin and these NOACs in real-world clinical practice allows opportunities for comparative effectiveness analyses, particularly of the gastrointestinal bleeding risk of these drugs when used outside the controlled setting of clinical trials. Because edoxaban was recently approved by the FDA in January 2015 and introduced to the market and because little real-world data are available, this study only focused on warfarin, dabigatran, rivaroxaban and apixaban. The main objective of our study was to compare the gastrointestinal bleeding risk among anticoagulated non-valvular atrial fibrillation patients on warfarin, dabigatran, rivaroxaban and apixaban. We compared GIB risk between each NOAC and warfarin, but also a direct pairwise comparison between individual NOACs. 

Real-world studies (RWSs), by integrating data from electronic health records, claims databases and disease registries, could extend findings of RCTs to large patient populations in real-world practice. The idealized settings of a clinical trial may not adequately reflect the real-world safety profile of NOACs as they are prescribed in routine clinical practice [54]. Therefore, RWSs are needed to clarify which anticoagulant would be the best choice for atrial fibrillation patients, to assess specifically the gastrointestinal safety profile.

**Figure 1:**
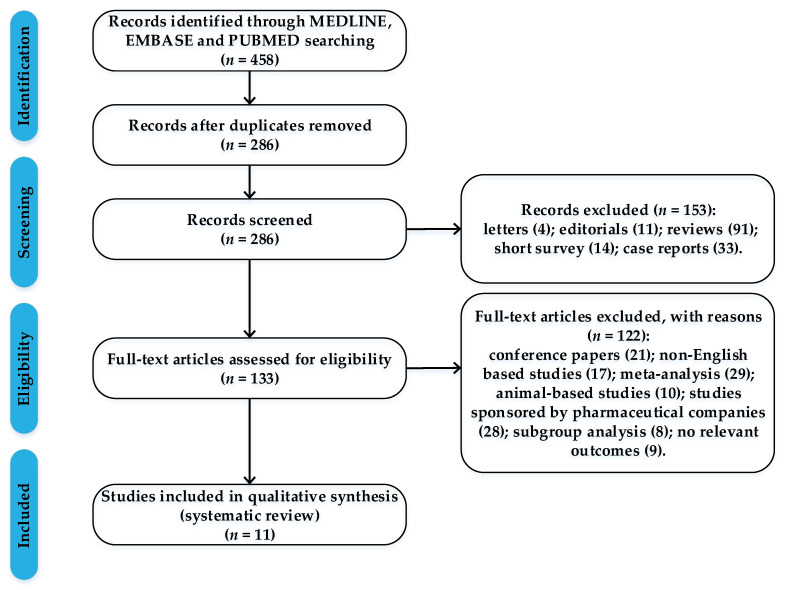
Flow chart with the process of article selection.

In the present study, we summarized evidence from high-quality RWSs for a comprehensive and rigorous analysis on the GIB risk for NOACs. We followed the PRISMA (preferred reporting items for systematic reviews and meta-analyses) guidelines when performing this research. These studies were selected by performing a systematic search of MEDLINE, EMBASE and PUBMED, using the following items: gastrointestinal bleeding risk, GIB, dabigatran, rivaroxaban, apixaban, warfarin, real-world studies, atrial fibrillation. We included in the research only high-quality real-world studies that fulfilled the following criteria: (1) reported major gastrointestinal bleeding events in patients given NOACs or warfarin; (2) available data on clinical events; (3) adjusted hazard ratios between each NOAC versus warfarin and from direct pairwise comparison of different NOACs for major gastrointestinal bleeding; (4) studies coordinated by independent research groups, published between 01 January 2017 and 31 December 2019. Considering that funding bias may be a form of publication bias, a phenomenon that is also recognized and studied by the researchers, we preferred to include only real-world data that were not sponsored by pharmaceutical companies. We excluded (1) animal-based studies; (2) non-English-based studies; (3) abstract, editorials, case reports and reviews (Figure 1).

Of 286 records screened, we included data from 11 high-quality real-world studies that reported major gastrointestinal bleeding events in patients given NOACs or vitamin K antagonists (Table 3). 

## 3. Results

### 3.1. Characteristics of Patients Included in the Studies

The primary outcome was major gastrointestinal bleeding, according to International Society on Thrombosis and Hemostasis criteria [64]. Bleeding was detected by upper endoscopy or colonoscopy, depending on the patient’s symptoms and personal pathological background. Data extracted from these studies included baseline characteristics, patient demographics, co-morbidities, CHA2DS2-VASC score, HAS-BLED score, pharmacologic risk factors (antiplatelet agents, non-steroidal anti-inflammatory drugs) for gastrointestinal bleeding and also data on major GI bleeding for patients with NOACs and vitamin K antagonists. After propensity-score matching, cohorts were closely balanced for all covariates, standardized differences of all baseline characteristics were <10% in all the studies, demonstrating similarity of comparators with regard to the important socio-demographic, co-morbidity and pharmacological risk factors (Table 4).

From all of the eleven studies, five of them evaluated the gastrointestinal bleeding risk by comparing each NOAC (dabigatran, rivaroxaban and apixaban) with warfarin, three by direct pairwise comparison of different NOACs and the other three by comparing each NOAC with warfarin and with each other NOAC.

### 3.2. Gastrointestinal Bleeding Risk Comparing Each NOAC with Warfarin

When comparing NOACs with warfarin, we found that in most studies the risk of major gastrointestinal bleeding, which accounted for more than 80% of major extracranial bleeding, was increased with dabigatran and rivaroxaban and decreased with apixaban, compared with warfarin. The lowest risk of gastrointestinal bleeding was with apixaban compared with warfarin (HR for gastrointestinal bleeding for apixaban ranged between 0.45 (95% CI 0.34 to 0.59) and 1.13 (95% CI 0.79 to 1.63)). Dabigatran was associated with lower or no significant difference in the risk of gastrointestinal bleeding (HR for dabigatran ranged between 0.58 (95% CI 0.47 to 0.71) and 1.43 (95% CI 1.07 to 1.90)) compared to warfarin use. Rivaroxaban had similar or even higher risk of gastrointestinal bleeding compared with warfarin (HR for rivaroxaban ranged between 1.00 (95% CI 0.87 to 1.16) and 1.38 (95% CI 1.12 to 1.54)) (Table 5).

It is surprising that the ranges of hazard ratios are so variable between the included studies, especially when comparing gastrointestinal bleeding risk of apixaban vs. warfarin (the ranges being between 0.45 to 1.13). The higher risk for gastrointestinal bleeding of each NOAC versus warfarin encountered during the first year in the study published by Forslund et al. [56] might be related to depletion of susceptibles, the imposibility of quantifying some factors, such as biological age or severity of the co-morbidities, and also the non-excluding design of the study, which included all new users of oral anticoagulants from primary health care, hospital based in- and outpatient care or specialized ambulatory care. Subgroup analyses highlighted similar outcomes with warfarin and NOAC treatment in high-risk patients aged 80 and above, and in patients with prior severe bleeds.

### 3.3. Gastrointestinal Bleeding Risk by Direct Pairwise Comparison of Different NOACs

Gastrointestinal bleeding occurred in all of the studies more frequently in patients given rivaroxaban than dabigatran (HR for rivaroxaban ranged between 1.15 (95% CI 0.99 to 1.36) and 1.35 (95% CI 0.91 to 2.00)). Apixaban was associated with a lower risk of GI bleeding than dabigatran (HR ranged between 0.39 (95% CI 0.27 to 0.58) and 0.95 (95% CI 0.65 to 1.18)) or rivaroxaban (HR ranged between 0.33 (95% CI 0.22 to 0.49) and 0.82 (95% CI 0.62 to 1.08)). We found that in all of the studies, apixaban had the most favorable GI safety profile and rivaroxaban the least favorable (Table 6).

## 4. Discussion

This study involves all available evidence from high-quality RWSs that were not supported by pharmaceutical companies for a comprehensive and rigorous analysis on the GIB risk for NOACs. The results of our study confirm a low or a similar risk for major GIB between patients receiving apixaban or dabigatran compared with warfarin, and apixaban appears to be associated with the lowest risk of GIB. In most of the studies included in our research, apixaban was associated with a significantly lower risk of gastrointestinal bleeding compared with warfarin. In a direct comparison of the gastrointestinal safety of the NOACs, apixaban appears to be associated with lower risk of gastrointestinal bleeding, whereas rivaroxaban is associated with higher risk of major gastrointestinal bleeding. The once-daily dosing of rivaroxaban and twice-daily administration of dabigatran and apixaban might also explain the higher risk of major bleeding in rivaroxaban, given its higher peak in plasma concentrations [65,66,67,68].

The possibility of a different bleeding site according to the type of molecule was discussed in only one study from the selected articles. Thus, in the study published by Vinogradova et al., it was observed that rivaroxaban was associated with higher risk compared with apixaban of a gastrointestinal and upper gastrointestinal bleed, in patients with atrial fibrillation. In those without atrial fibrillation, apixaban was associated with a lower risk of a gastrointestinal and upper gastrointestinal bleed compared with warfarin. Dabigatran and rivaroxaban were associated with a higher risk of a gastrointestinal bleed compared with apixaban, and rivaroxaban was also associated with a higher risk of an upper gastrointestinal bleed [62].

Up to now, there have been systematic reviews and metaanalyses conducted to assess the gastrointestinal risk of NOACs, but from our knowledge, there is no review that has included only real world studies that were not supported by pharmaceutical companies.

Among different NOACs, it is difficult to conclude which drug has the lowest gastrointestinal bleeding risk as there are no direct head-to-head comparisons in randomized clinical trials (RCTs) and because patient characteristics are different across studies. Up to now, several systematic reviews and metaanalyses have been conducted to assess the GIB risk of NOACs in randomized clinical trials. One of the earliest meta-analyses [69], which included 17 RCTs with a total of 75,081 patients who received either a NOAC or standard care (either low-molecular-weight heparin, vitamin K antagonist, antiplatelet therapy or placebo), demonstrated a 1.5% GIB event, with 89% being major GIB (defined as GIB leading to a decrease in hemoglobin ≥ 2 g/dl within 24 h, a transfusion of ≥ 2 units of packed red cells, necessitating intervention including surgery, or fatal bleeding). Holster et al. also demonstrated that there was an increased risk of GIB among NOAC users compared with standard care and that among different NOACs, dabigatran and rivaroxaban were associated with a higher risk of GIB (OR 1.58 and 1.48, respectively) [69].

In a recent systematic review and meta-analysis of data from randomized controlled trials and real-world studies, Gu et al. [70] evaluated the risk of major gastrointestinal bleeding in patients with NOACs compared with conventional treatment and confirmed that there is no significant difference between these two groups. They analyzed data from 43 randomized controlled trials (183,752 patients) and 41 real-world studies (1,879,428 patients). The pooled major rates of GIB for patients on NOACs (1.19%) vs conventional treatment (0.92%) did not differ significantly (HR from real-world studies, 1.02; 95% CI, 0.94–1.10; P interaction = 0.52). Rivaroxaban, but not other NOACs, was associated with an increased risk of major GIB (HR from real-world studies, 1.14; 95% CI, 1.04–1.23; P interaction = 0.06). Rivaroxaban users had a 39% increase in risk for major GIB. Analyses of subgroups, such as patients with different indications, dosage, or follow-up time, did not significantly affect results.

In another systematic review and meta-analysis, Li et al. [71] summarized the evidence of observational studies for direct comparative safety amongst NOACs in patients with atrial fibrillation. They included fifteen studies for qualitative synthesis and twelve studies for meta-analyses. Rivaroxaban was associated with a significantly higher risk of major bleeding, in comparison with apixaban (HR = 1.71, 95% CI 1.51–1.94; evidence quality: low). Compared with dabigatran, apixaban was associated with lower risk of major bleeding (HR = 0.80, 95% CI 0.68–0.95; evidence quality: low). Apixaban was associated with a lower risk of major bleeding when compared with dabigatran or rivaroxaban, so it was found to have the most favorable safety profile amongst the three NOACs.

The major strength of this study was to reassess the risk for major GIB of NOACs by comparing the results from high-quality RWSs that were not supported by pharmaceutical companies. Certainly there are inherent limitations in this study. First, we included only 11 studies that met the criteria, which may reduce the statistical power. Second, all included studies reported major GIB events according to the International Society on Thrombosis and Hemostasis criteria, but it is not possible to evaluate whether small variations may have an impact on the results obtained. Another weakness of our study is that we did not perform a meta-analysis and, consequently, there are not any pooled estimates of hazard or risk ratios. In addition, we did not have the resources to review non-English articles, but we are confident that this study covered the majority of high-quality RWSs identified in a comprehensive search of broad databases.

## 5. Conclusions

The results of our study from real-world studies confirm a low or a similar risk for major gastrointestinal bleeding between patients receiving NOACs compared with conventional treatment with warfarin. In a direct comparison of the gastrointestinal safety of the NOACs, apixaban appears to be associated with a lower risk of gastrointestinal bleeding, whereas rivaroxaban is associated with a higher risk of major gastrointestinal bleeding. Our findings may provide some decision-making support for physicians regarding their selection of oral anticoagulant treatment based on risk for major gastrointestinal bleeding in patients with AF.

## Figures and Tables

**Table 1:** Indications and dosing of NOACs in Europe.

Indication	Dabigatran	Apixaban	Rivaroxaban
Non-valvular atrial fibrillation	150 mg b.i.d	5 mg b.i.d	20 mg daily
	110 mg b.i.d if age ≥ 80 years (may consider 110 mg b.i.d also if increased risk of bleeding)	2.5 mg b.i.d if CrCl 15–29 mL/min OR two out of the following: age ≥ 80 years, BW ≤ 60 kg, Cr ≥ 1.5 mg/dL	-
	Avoid if CrCl < 30 mL/min	Avoid if CrCl < 15 mL/min	Avoid if CrCl < 15 mL/min
Treatment and prevention of recurrent deep vein thrombosis/ pulmonary embolism	150 mg b.i.d after 5 days of initial therapy with a parenteral anticoagulant	10 mg b.i.d for 1 week, then 5 mg b.i.d	15 mg b.i.d for 3 weeks, then 20 mg daily
	110 mg b.i.d after 5 days of initial therapy with a parenteral anticoagulant if age ≥ 80 years (may consider 110 mg b.i.d also if increased risk of bleeding)	-	-
	Avoid if CrCl < 30 mL/min	Avoid if CrCl < 15 mL/min	Avoid if CrCl < 15 mL/min

NOACs: non-vitamin K antagonist oral anticoagulants; CrCl: Creatinine clearance; BW: Body weight; Cr: Creatinine; b.i.d: Twice a day.

**Table 2:** Factors associated with NOACs-related gastrointestinal bleeding.

**Risk Factors**
1. Higher dose of dabigatran: a dose of 150 mg b.i.d 2. Concomitant use of ulcerogenic agents like antiplatelet agents, non-steroidal anti-inflammatory drugs or steroid 3. Older age: ≥75 years 4. Renal impairment with a creatinine clearance <50 mL/min 5. Prior history of peptic ulcers or GIB 6. Helicobacter pylori infection 7. Pre-existing GI tract lesions such as: diverticulosis, angiodysplasias 8. Ethnicity: western population 9. HAS-BLED score ≥3
**Protective Factors**
Gastroprotective agents: proton pump inhibitors or histamine H2-receptor antagonists

HAS-BLED: a scoring system developed to assess 1-year risk of major bleeding in patients taking anticoagulants with atrial fibrillation. The score is between 0 and 9, based on seven parameters: hypertension, abnormal renal/liver function (1 point each), stroke, bleeding history or predisposition, labile INR, elderly (>65 years), drug/alcohol concomitantly (1 point each).

**Table 3:** Real-world studies that analyzed NOACs-related gastrointestinal bleeding.

Study	Country	Obser-Vation Period	Oral Anticoagu-Lants Analyzed	Methodology	Population	Endpoint
Abraham et al. [54]	USA	October 2010 to February 2015	Apixaban, dabigatran or rivaroxaban	Retrospective cohort study based on medical and pharmacy claims data from OptumLabs Data Warehouse using PSM	43,303 adults diagnosed with NVAF who had an indexprescription for apixaban, dabigatran, or rivaroxaban	Bleeding
Adeboyeje et al. [55]	USA	November 2010 to February 2015	Apixaban, dabigatran, rivaroxaban, warfarin	Retrospective cohort study based on data from a commercially insured population in the U.S.	44,057 patients diagnosed with NAVF, who used warfarin (23,431), dabigatran (8539), apixaban (3689) and rivaroxaban (8398)	Bleeding
Andersson et al. [30]	Denmark	July 2013 to March 2016	Apixaban, dabigatran or rivaroxaban	Retrospective cohort study based on nationwide registers	12,638 NVAF patients, propensity scores in a 1:1 ration comparing (apixaban vs. dabigatran = 6470; apixaban vs. rivaroxaban = 7352; rivaroxaban vs. dabigatran = 5440 patients)	Effective-ness + bleeding
Forslund et al. [56]	Sweden	January 2012 to December 2015	Apixaban, dabigatran, rivaroxaban, warfarin	Population-based retrospective cohort study based on data from the Stockholm Region administrative health data register containing healthcare utilization and prescription data	22,198 adults with NVAF who were naive for either warfarin or one of the following NOACs: apixaban, dabigatran, or rivaroxaban during the study period.	Effective-ness + bleeding
Graham et al. [57]	USA	October 2010 to September 2015	Apixaban, dabigatran, rivaroxaban, warfarin	Retrospective new-users cohort study based on data from Medicare beneficiaries	183,318 warfarin, 86,198 dabigatran, 106,389 rivaroxaban and 73,039 apixaban users	Effective-ness + bleeding
Hernandez et al. [58]	USA	January 2013 to December 2014	Apixaban, dabigatran, rivaroxaban, warfarin	Retrospective database analysis from a random sample of Medicare beneficiaries	41,366 adults with NVAF who were naive for either warfarin or one of the following NOACs: apixaban, dabigatran or rivaroxaban during the study period.	Effective-ness + bleeding
Larsen et al. [59]	Denmark	August 2011 to October 2015	Warfarin, apixaban, dabigatran, rivaroxaban (only standard dose)	Retrospective database analysis using propensity score weighting (inverse probability of treatment weighting)	61,678 NVAF patients, naive to OAC, first time on DOAC or warfarin	Effective-ness + bleeding
Nielsen et al. [60]	Denmark	August 2011 to February 2016	Warfarin, apixaban, dabigatran, rivaroxaban (only reduced dose)	Retrospective analysis using propensity score weighting (inverse probability of treatment weighting)	55,644 NVAF patients, naïve to OAC, first time on DOAC or warfarin, all restricted to reduced dose	Effective-ness + bleeding
Noseworthy et al. [61]	USA	October 2010 to June 2015	Rivaroxaban, dabigatran, apixaban	Retrospective analysis using administrative claims, using PSM and Cox proportional hazards regression	57,788 NVAF patients, including patients with prior warfarin exposure. Apixaban used as reference category	Effective-ness + bleeding
Vinogradova et al. [62]	UK	January 2011 to October 2016	Warfarin, apixaban, dabigatran, rivaroxaban	Prospective open cohort study using two primary care databases representative of the national population	132,231 warfarin, 7744 dabigatran, 37,863 rivaroxaban and 18,223 apixaban users, subgrouped into 103,270 patients with atrial fibrillation and 92,791 without atrial fibrillation	Bleeding
Yao et al. [63]	USA	October 2010 to June 2015	Warfarin, apixaban, dabigatran, rivaroxaban	Retrospective database analyses using PSM	125,243 NVAF patients, three 1:1 PSM cohorts (apixaban = 15,390; dabigatran = 28,614; rivaroxaban = 32,350)	Effective-ness + bleeding

NOACs: non-vitamin K antagonist oral anticoagulants; NVAF: nonvalvular atrial fibrillation; OAC: oral anticoagulant; DOAC: direct oral anticoagulant; PSM: propensity score matching.

**Table 4:** Baseline characteristics of patients included in the studies.

	Dabigatran	Rivaroxaban	Apixaban	Warfarin	Maximum SMD
**Study Abraham et al. [54]**					
Age group (mean)	68.8 (11.4)	70.6 (11.4)	72.3 (11.1)	NA	-
Male	60.5%	57.1%	54%	NA	-
CHA2DS2-VASC (mean)	3.6 (1.9)	3.8 (1.9)	4.0 (1.9)	NA	-
HAS-BLED (mean)	2.2 (1.2)	2.4 (1.2)	2.4 (1.2)	NA	-
Antiplatelet or non-steroidal anti-inflammatory drugs	10.7%	12.1%	12.3%	NA	-
**Study Adeboyeje et al. [55]**					
Age group (mean)	70 (12.3)	70 (12.3)	70 (12.6)	70 (12.2)	0.01
Male	58.9%	58.7%	59.5%	59.1%	0.01
CHA2DS2-VASC (mean)	3.3 (1.9)	3.3 (1.9)	3.3 (1.9)	3.3 (1.8)	0.01
HAS-BLED (mean)	2.1 (1.4)	2.1 (1.4)	2.1 (1.4)	2.1 (1.4)	0.01
Antiplatelet or non-steroidal anti-inflammatory drugs	19.9%	20.5%	20.2%	20.2%	0.01
**Study Andersson et al. [30]**					
Age group (mean)	65.7 (7.3)	72.0 (9.8)	71.9 (9.1)	NA	-
Male	64%	56%	63%	NA	-
CHA2DS2-VASC	-	-	-	NA	-
HAS-BLED	-	-	-	NA	-
Antiplatelet or non-steroidal anti-inflammatory drugs	59.8%	61.8%	57.8%	NA	-
**Study Forslund et al. [56]**					
Age group (mean)	69.9 (11.3)	74.0 (10.3)	75.0 (10.8)	74.1 (11.0)	-
Male	60%	54.6%	54.6%	55.4%	-
CHA2DS2-VASC (mean)	3. 01 (1.89)	3.59 (1.88)	3.69 (1.9)	3.68 (1.91)	-
HAS-BLED	-	-	-	-	-
Antiplatelet drugs	46.73%	55.74%	47.73%	54.96%	-
**Study Graham et al. [57]**					
Age group (mean)	75.5	74.9	75.2	75.8	0.15
Male	52.4%	53.9%	52.2%	52%	0.04
CHA2DS2-VASC (≥2)	96.7%	96.6%	97.4%	97.1%	0.04
HAS-BLED (≥3)	44.7%	43.7%	47.8%	45.8%	0.03
Antiplatelet or non-steroidal anti-inflammatory drugs	28.3%	28.2%	29.5%	27.2%	0.04
**Study Hernandez et al. [58]**					
Age group (mean)	74.9 (8.7)	76.4 (8.6)	77.4 (8.6)	76.0 (10.3)	<0.001
Male	47.0%	43.7%	42.5%	43.1%	<0.001
CHA2DS2-VASC (mean)	4.26 (1.74)	4.55 (1.78)	4.68 (1.73)	4.8 (1.82)	<0.001
HAS-BLED (mean)	3.49 (0.93)	3.65 (0.95)	3.71 (0.93)	3.71 (1.0)	<0.001
Antiplatelet or non-steroidal anti-inflammatory drugs	22.5%	25.2%	25.0%	21.3%	<0.001
**Study Larsen et al. [59]**					
Age group (mean)	67.6 (5.6)	71.8 (7.1)	71.3 (5.9)	72.4 (7.4)	0.02
Male	66.1%	57.9%	60.3%	58.8%	0.02
CHA2DS2-VASC (mean)	2.2 (1.4)	2.8 (1.6)	2.8 (1.6)	2.8 (1.7)	0.02
HAS-BLED (mean)	2.0 (1.1)	2.2 (1.2)	2.3 (1.2)	2.2 (1.2)	0.01
Aspirin or non-steroidal anti-inflammatory drugs	62.7%	60.4%	60.2%	66.3%	0.01
**Study Nielsen et al. [60]**					
Age group (mean)	79.9 (9.0)	77.9 (13.5)	83.9 (8.2)	71.0 (12.6)	0.09
Male	46.3%	46.8%	39.4%	59.6%	0.03
CHA2DS2-VASC (mean)	3.8 (1.5)	3.6 (1.8)	4.3 (1.5)	3.0 (1.7)	0.04
HAS-BLED (mean)	2.7 (1.0)	2.5 (1.2)	2.8 (1.1)	2.4 (1.2)	0.06
Aspirin or non-steroidal anti-inflammatory drugs	74.8%	66.2%	66.7%	70.2%	0.03
**Study Noseworthy et al. [61]**					
Age group (median)	71 (62–78)	73 (65–81)	73 (65–81)	NA	-
Male	58.9%	54.4%	54.1%	NA	-
CHA2DS2-VASC (median)	4 (2–5)	4 (3–5)	4 (3–5)	NA	-
HAS-BLED (median)	2 (1–3)	2 (2–3)	2 (2–3)	NA	-
Antiplatelet or non-steroidal anti-inflammatory drugs	11.1%	11.7%	12.2%	NA	-
**Study Vinogradova et al. [62]**					
Age group (mean)	74.5 (10.7)	75.8 (10.8)	76.5 (10.9)	74.8 (10.4)	-
Male	59.5%	54.3%	53.4%	55.6%	-
CHA2DS2-VASC	-	-	-	-	-
HAS-BLED	-	-	-	-	-
Antiplatelet or non-steroidal anti-inflammatory drugs	39.6%	32.3%	31.3%	41.8%	-
**Study Yao et al. [63]**					
Age group (median)	70 (62–78)	72 (64–80)	73 (66–81)	73 (66–81)	-
Male	61.3%	57.8%	53.1%	53.2%	-
CHA2DS2-VASC (median)	3 (2–5)	4 (2–5)	4 (3–5)	4 (3–5)	-
HAS-BLED (median)	2 (1–3)	2 (2–3)	2 (2–3)	2 (2–3)	-
Antiplatelet or non-steroidal anti-inflammatory drugs	10.3%	11.6%	12.1%	12.5%	-

SMD—standardized mean difference; CHA2DS2-VASC score—assigns points for the presence of congestive heart failure, hypertension, age 65–74 years and age ≥75 years, diabetes mellitus, stroke or transient ischemic attack, vascular disease, and female sex; HAS-BLED score—assigns points for the presence of hypertension, abnormal renal or liver function, stroke, bleeding history, labile INR, age ≥65 years, and antiplatelet drug or alcohol use; NA—not applicable.

**Table 5:** Adjusted hazard ratios with 95% confidence intervals from comparisons of each NOAC versus warfarin for major gastrointestinal bleeding.

Study	Dabigatran vs. Warfarin	Rivaroxaban vs. Warfarin	Apixaban vs. Warfarin
Forslund et al. [56]	1.43 (1.07–1.9)	1.28 (0.90–1.80)	1.13 (0.79–1.63)
Larsen et al. [59]	0.58 (0.47–0.71)	1.06 (0.91–1.23)	0.61 (0.49–0.75)
Nielsen et al. [60]	0.87 (0.75–1.01)	1.17 (0.94–1.45)	1.04 (0.76–1.43)
Vinogradova et al. [62]	1.08 (0.83–1.41)	1.21 (1.01–1.45)	0.76 (0.58–0.99)
Yao et al. [63]	0.79 (0.67–0.94)	1.04 (0.90–1.20)	0.45 (0.34–0.59)
Graham et al. [57]	1.04 (0.9–1.21)	1.38 (1.12–1.54)	0.51 (0.42–0.61)
Adeboyeje et al. [55]	1.17 (1.04–1.32)	1.00 (0.87–1.16)	0.82 (0.63–1.06)
Hernandez et al. [58]	0.95 (0.75–1.19)	1.35 (1.20–1.52)	0.72 (0.57–0.90)

**Table 6:** Adjusted hazard ratios with 95% confidence intervals from direct pairwise comparison of different NOACs for major gastrointestinal bleeding.

Study	Rivaroxaban vs. Dabigatran	Apixaban vs. Dabigatran	Apixaban vs. Rivaroxaban
Andersson et al. [30]	1.35 (0.91–2.00)	0.94 (0.62–1.41)	0.88 (0.64–1.22)
Abraham et al. [54]	1.20 (1.00–1.45)	0.39 (0.27–0.58)	0.33 (0.22–0.49)
Noseworthy et al. [61]	1.30 (1.10–1.53)	0.50 (0.36–0.70)	0.39 (0.28–0.54)
Graham et al. [57]	1.32 (1.21–1.45)	0.56 (0.32–0.74)	0.38 (0.27–0.59)
Adeboyeje et al. [55]	1.15 (0.99–1.36)	0.95 (0.65–1.18)	0.82 (0.62–1.08)
Hernandez et al. [58]	1.25 (1.06–1.39)	0.76 (0.56–1.03)	0.53 (0.42–0.68)
